# Periosteum Metabolism and Nerve Fiber Positioning Depend on Interactions between Osteoblasts and Peripheral Innervation in Rat Mandible

**DOI:** 10.1371/journal.pone.0140848

**Published:** 2015-10-28

**Authors:** Cédric Mauprivez, Caroline Bataille, Brigitte Baroukh, Annie Llorens, Julie Lesieur, Pierre J. Marie, Jean-Louis Saffar, Martin Biosse Duplan, Marc Cherruau

**Affiliations:** 1 EA2496 Laboratoire Pathologies, Imagerie et Biothérapies oro-faciales, Faculté de Chirurgie Dentaire, Université Paris Descartes, Sorbonne Paris Cité, 1 rue Maurice Arnoux 92120, Montrouge, France; 2 Assistance Publique – Hôpitaux de Paris, Avenue Victoria, Paris, France; 3 UMR-1132 INSERM and Université Paris Diderot, Sorbonne Paris Cité, Hôpital Lariboisière, Paris, France; 4 INSERM U1163, Université Paris Descartes, Sorbonne Paris Cité, Institut Imagine, Hôpital Necker-Enfants Malades, Paris, France; Université de Lyon - Université Jean Monnet, FRANCE

## Abstract

The sympathetic nervous system controls bone remodeling by regulating bone formation and resorption. How nerves and bone cells influence each other remains elusive. Here we modulated the content or activity of the neuropeptide Vasoactive Intestinal Peptide to investigate nerve-bone cell interplays in the mandible periosteum by assessing factors involved in nerve and bone behaviors. Young adult rats were chemically sympathectomized or treated with Vasoactive Intestinal Peptide or Vasoactive Intestinal Peptide_10-28_, a receptor antagonist. Sympathectomy depleted the osteogenic layer of the periosteum in neurotrophic proNerve Growth Factor and neurorepulsive semaphorin3a; sensory Calcitonin-Gene Related Peptide-positive fibers invaded this layer physiologically devoid of sensory fibers. In the periosteum non-osteogenic layer, sympathectomy activated mast cells to release mature Nerve Growth Factor while Calcitonin-Gene Related Peptide-positive fibers increased. Vasoactive Intestinal Peptide treatment reversed sympathectomy effects. Treating intact animals with Vasoactive Intestinal Peptide increased proNerve Growth Factor expression and stabilized mast cells. Vasoactive Intestinal Peptide_10-28_ treatment mimicked sympathectomy effects. Our data suggest that sympathetic Vasoactive Intestinal Peptide modulate the interactions between nervous fibers and bone cells by tuning expressions by osteogenic cells of factors responsible for mandible periosteum maintenance while osteogenic cells keep nervous fibers at a distance from the bone surface.

## Introduction

Bone metabolism results from a complex and subtle interplay of systemic and local actors that intervene on the differentiation and activities of osteoclasts, osteoblasts and osteocytes that interact to fine tune and couple remodeling activities. These interactions are locally mediated by numerous factors among which the RANK-RANKL-OPG triad has a prominent role [1].

The nervous system has emerged as an important regulator of bone metabolism by centrally controlling bone cell activities. Its peripheral effector, the adrenergic sympathetic nervous system, controls bone formation and resorption mostly through the ß2-adrenergic receptors (AdB2R) in the appendicular and axial skeletons [2]. AdB2R activation up-regulates RANKL (an activator of resorption) by osteoblasts [3]. Whether bone cells exert any control on the adjacent nervous fibers, remains elusive. In adult animals, nerve growth factor (NGF) released by left ventricular cardiomyocytes modulates heart sympathetic innervation: enhanced NGF expression increases innervation while NGF depletion results in sympathetic hypoinnervation [4]. During sweat gland development, the postganglionic sympathetic neurons expressing a dual cholinergic/adrenergic phenotype are committed to express the sole cholinergic phenotype by factors released by the target [5]. In the bone environment, osteoblasts switch the phenotype of sympathetic fibers from an adrenergic to a cholinergic phenotype during the establishment of sternum periosteum innervation [6,7]. However, little is known on the reciprocal influences between bone cells and nervous fibers.

Attractant (or trophic) and repulsive molecules guide axons during development and tune tissue innervation plasticity in the adulthood. Noticeably, some of these agents are also involved in bone metabolism. NGF is an attraction factor, member of the neurotrophin family. In adults, NGF is involved in nerve fiber maintenance and plasticity, in induction or inhibition of neuropeptides and neurotransmitters [8]. The target tissues of NGF-dependent sympathetic and sensory neurons release NGF in concentration proportional to nerve fiber density [9]. NGF signals through two receptors, the cognate receptor, tyrosine kinase receptor A (TrkA), and the p75 pan neurotrophin receptor, a low affinity non-selective receptor. Osteoblastic cells constitutively express NGF [10,11]. NGF activates the differentiation of mouse calvaria osteoblasts [12], behaves as an autocrine anti-apoptotic factor [10] and enhances callus maturation and mineral apposition after mandible distraction osteogenesis [13].

Semaphorin 3A (sema3a), a repulsive molecule for sensory and sympathetic neurons [14,15], is involved in axon guidance and in fiber plasticity in adults [16]. Sema3a exerts its biological activity through interactions with the neuropilin-1 (NRP1) receptor complexed with class A plexin receptor for signaling [17]. In adults, sema3a inhibits fiber sprouting. Changes in sema3a expression induce reactive sprouting and abnormal connections in the central [16] and peripheral [18] nervous systems. Osteoblasts and osteocytes express sema3a mRNA [11]. Sema3a stimulates bone mass by enhancing osteoblast differentiation and by inhibiting osteoclast differentiation [19]. Sema3a also inhibits monocyte migration [20]. As a whole, besides acting on the maintenance of the sympathetic and sensory nervous networks, both NGF and sema3a participate in the control of bone metabolism.

In the mandible periosteum the sympathetic nervous system modulates bone metabolism through its cholinergic (and not adrenergic) pathway. Chemical sympathectomy increases OPG expression, inhibits preosteoclast differentiation [21] and bone resorption [22,23]. Treatment of sympathectomized animals with vasoactive intestinal peptide (VIP), a pleiotropic peptide with neuroprotective actions, restores the resorption potential of the periosteum [21]. Anatomically, the VIP-immunoreactive (IR) fibers are located at the boundary of the osteogenic and fibrous compartments of the periosteum [21,23]. While these data show that the cholinergic innervation significantly impacts osteoblastic cell behavior, how these cells act on the fibers innervating the periosteum remains unknown. The aim of this study was to determine in the mandible periosteum the interrelationships of cells of the osteoblastic lineage and the nervous fibers present in their environment. To this goal, VIP content and/or activity were modulated in vivo and the impact on osteoblast activity and expression of NGF and sema3a was determined.

## Materials and Methods

Eight weeks-old male Wistar rats (Iffa Credo, L’Arbresle, France), weighing 250 ± 20 g, fed a standard rodent diet (M25 Extralabo; UAR, Villemoisson, France) were used. Food and water were provided ad libitum. The animals were maintained in a temperature-controlled (25°C) facility with a 12 h light/dark cycle. Animal condition was monitored daily. Before euthanasia, rats were anesthetized with 8% chloral solution (Prolabo, Fontenay, France), a thoracostomy was performed and the animals were killed by cardiac exsanguination. The study was performed in keeping with European Community recommendations on laboratory animal care (decree 87–848–04/19/1987) Our local facilities are accredited (French Veterinary Service license #C92-049-01) and JLS and BB hold a license to experiment on live animals (level 1, French Ministère de l’Agriculture, licenses #92–205, #92–353, respectively). The experiments were performed before January 1, 2013 (i.e. before approval by an ethical committee was mandatory in France), so no animal ethics committee was consulted; however the study was under the control of Anima 5, the coordination committee for animal housing facilities at University Paris Descartes that enacted guidelines for animal experiments before this date. The protocols only included injections that did not necessitate any analgesic treatment. No animal died during the experimental periods. Observations of the animals did not evidence changes in health or behavior, or discomfort, whatever the treatment.

### Time-course study of chemical sympathectomy

Forty 8 weeks-old rats were distributed at random in two lots. Twenty rats were intraperitoneally (ip) injected daily with guanethidine monosulfate (Zhou Fang Pharm Chemical, Shanghai, China; 40 mg/kg/day in saline), a neurotoxic for rat sympathetic neurons [24]. Twenty rats were sham-treated with daily saline ip injections and used as controls. Ten control and sympathectomized rats were killed 7 and 17 days after starting the treatment [21]. Ptosis appeared in all sympathectomized rats as early as day 3 after starting the guanethidine treatment, a precocious sign of sympathectomy onset and effectiveness as previously described [23].

### Effects of VIP and of VIP_10-28_ treatments

Forty other rats were used. Ten rats were sympathectomized for 17 days as previously and then ip injected with VIP (NeoMPS, Strasbourg, France; 25 nmol/kg/d in saline) for 10 additional days. Three other groups of 10 intact, i.e. not sympathectomized, rats were ip injected with either VIP (25 nmol/kg/d in saline) or VIP_10-28_, a non-selective VIP receptor antagonist (25 nmol/kg/d in saline, American Peptide Company, Sunnydale, CA, USA) or saline for 10 days.

### Sample Processing

At the end of their respective treatment periods, the rats were killed by cardiac exsanguination. The mandibles were removed and fixed for 24 hours at 4°C in either 70% ethanol (right hemi-mandibles) or Zamboni’s fixative (4% paraformaldehyde and 3% picric acid in phosphate buffer, pH 7.2–7.4; left hemi-mandibles). After rinsing and dehydration in ascending ethanol solutions, the bones were embedded without demineralization at -20°C in methyl methacrylate (Merck, Darmstadt, Germany). Four micron-thick sections were cut in the horizontal plane (i.e. perpendicular to the molar root axis) using a Polycut E microtome (Leica, Wetzlar, Germany). Three consecutive series of 10 serial sections were taken from the top of the crest of the alveolar process as described previously [23]. The sections were sequentially stained with toluidine blue (pH 2.5), or processed for alkaline phosphatase (ALP) enzymohistochemistry and for immunohistochemistry. The 5^th^ section of each series was processed for ALP that was detected by incubating the sections with naphthol ASTR phosphate and fast blue RR (pH 9) to reveal osteogenic cells (preosteoblasts and osteoblasts). Each marker staining was assigned a position within the series on either side of the ALP-stained section, so that two sections stained for a marker were 40 μm apart.

To standardize periosteum physiological conditions, animals with osteoclastic resorption along the buccal cortex were discarded, as this may modify the expression profile of the different cell types.

### Immunochemistry

NGF is transcripted as pre-proNGF, cleaved in the endoplasmic reticulum into proNGF before transiting to the Golgi apparatus from where it is stored and cleaved within secretory vesicles into mature NGF (ßNGF), or excreted as proNGF and extracellularly processed [25,26]. ProNGF is biologically active [27]. Since these two pathways may be involved, we looked for proNGF and ßNGF expressions.

Monoclonal rabbit anti-ßNGF (04–1119, Millipore, Temecula, CA) and polyclonal [rabbit anti-proNGF (sc548, Santa Cruz Biotechnologies, Santa Cruz, CA, USA), anti-TrkA (sc118, Santa Cruz Biotechnology), anti-semaphorin3a (ab23393, Abcam, Cambridge, UK), anti-CGRP (C8198, Sigma, St Louis, MO, USA), anti-neuropilin-1 (LS-C148411, Life Span Biosciences, Seattle, WA, USA), anti-substance P (AB1566, Chemicon, Temicula, CA, USA) and goat anti-tissue plasminogen activator (tPA, ab14198, Abcam)] antibodies were used. Ten percent normal horse serum (Eurobio, Les Ulis, France) (monoclonal antibody), normal goat or rabbit sera (Eurobio) (polyclonal antibodies) in 0.1M PBS with 0.05% Tween 20 were used to reduce nonspecific background. The sections were incubated overnight with primary antibodies and then with the relevant secondary biotinylated antibody (Vector, Burlingame, CA, USA) with 3% hydrogen peroxide and an avidin-biotin peroxidase complex (ABC Vectastain kit, Vector). Diaminobenzidine tetrahydrochloride (Sigma) was the chromogen. Negative controls were prepared by omitting the primary antibody, by replacing the primary antibody with nonimmune serum at the same dilution, or by using an irrelevant secondary antibody.

To ensure reproducibility of the measurements of each experimental step, the sections immunostained for a marker were processed together.

### Histomorphometry

**Fig 1:**
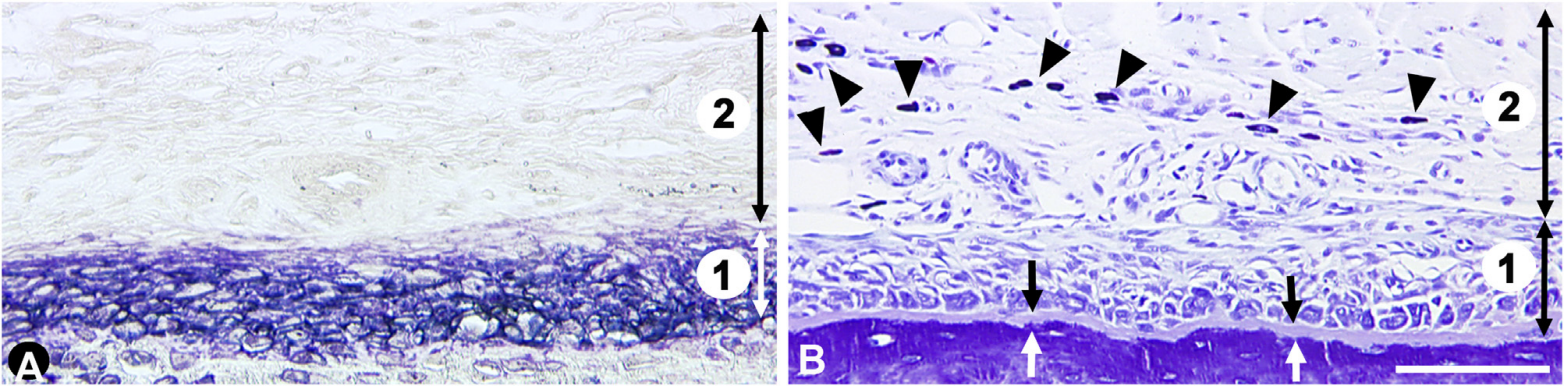
General view of the mandible periosteum from control animals. **A.** Enzymohistochemistry staining for alkaline phosphatase revealing the cells of the osteogenic lineage. This layer was used to delineate the osteogenic compartment (zone 1) from the non-osteogenic compartment (zone 2). **B.** Toluidine blue staining. The arrowheads point out the mast cells present in the non-osteogenic compartment (2). At the interface with the mineralized bone matrix, the arrows delineate the osteoid seam. The double arrows separate the two compartments. Same magnification in **A** and **B**, bar: 100 μm.

The sections were examined morphometrically at a constant magnification (x250) with a semiautomatic image analyzer along the mandible buccal periosteum. The zone under study extended from the distal aspect of the mesial root to the distal aspect of the distal root of the first molar. The ALP staining revealed a thick layer of ALP^+^ osteogenic cells, separated from the mineralized bone surface by a layer of osteoid tissue (Fig 1A and 1B).

The ALP layer was used as a reference to divide the periosteal zone into juxta-osseous osteogenic and outer non-osteogenic compartments (Fig 1A and 1B). The ALP-positive layer was outlined on the computer screen and recorded. It was then superimposed on each section of the relevant series. This allowed us to visualize the location of the ALP layer in all the specifically stained sections. The measurements were made on 2 sections for each marker. The following parameters were recorded in the osteogenic compartment: (a) length of the reference bone segment; (b) area of the osteogenic layer (in mm^2^); (c) thickness of the osteoid seam (in μm); (d) proNGF immunostaining in the osteogenic layer, expressed as a percentage of proNGF immunostaining per osteogenic layer area; (e) number of proNGF-IR fibers per mm of adjacent bone surface (N/mm); (f) thickness of the proNGF immunostaining within the osteoid seam (in μm). As we observed that only a portion of the osteoid seam was immunostained, we calculated the fraction of the osteoid seam immunopositive for proNGF (proNGF thickness/osteoid thickness ratio); (g) percentage of TrkA immunostaining per osteogenic layer area; (h) number of sema3a^+^ cells in the osteogenic layer, expressed in cells per mm of adjacent bone surface (N/mm): (i) number of sema3a^+^ osteocytes present in a 60 μm-deep layer of bone matrix subjacent to the reference bone segment surface (N/mm of adjacent bone surface); (j) number of CGRP immunopositive spots that included both osteoblastic CGRP expression and sensory IR fibers (N/mm^2^). The procedure was as follows: the software was set at the specific color of CGRP immunostaining and the computer automatically detected the pixels corresponding to this color. We verified the location of sensory fibers by performing immunostaining for substance P, another marker of sensory fibers.

In the non-osteogenic compartment, we quantified (a) the number of ßNGF^+^ mast cells (N/mm of adjacent bone surface). Total and activated mast cells were counted separately according to Fouilloux et al. [29] and the activated/total cells ratio was calculated; (b) number of CGRP immunopositive spots that included only sensory IR fibers (N/mm^2^).

### Statistics

The data were compared (7 samples per group) using nonparametric tests (Kruskal—Wallis test followed, if significant, by group comparisons with the Mann—Whitney *U*-test). Differences were considered significant at *p* ≤ 0.05. Data are expressed as mean ± SEM.

## Results

### Effects of sympathectomy in the osteogenic compartment

#### NGF and TrkA expression

**Fig 2:**
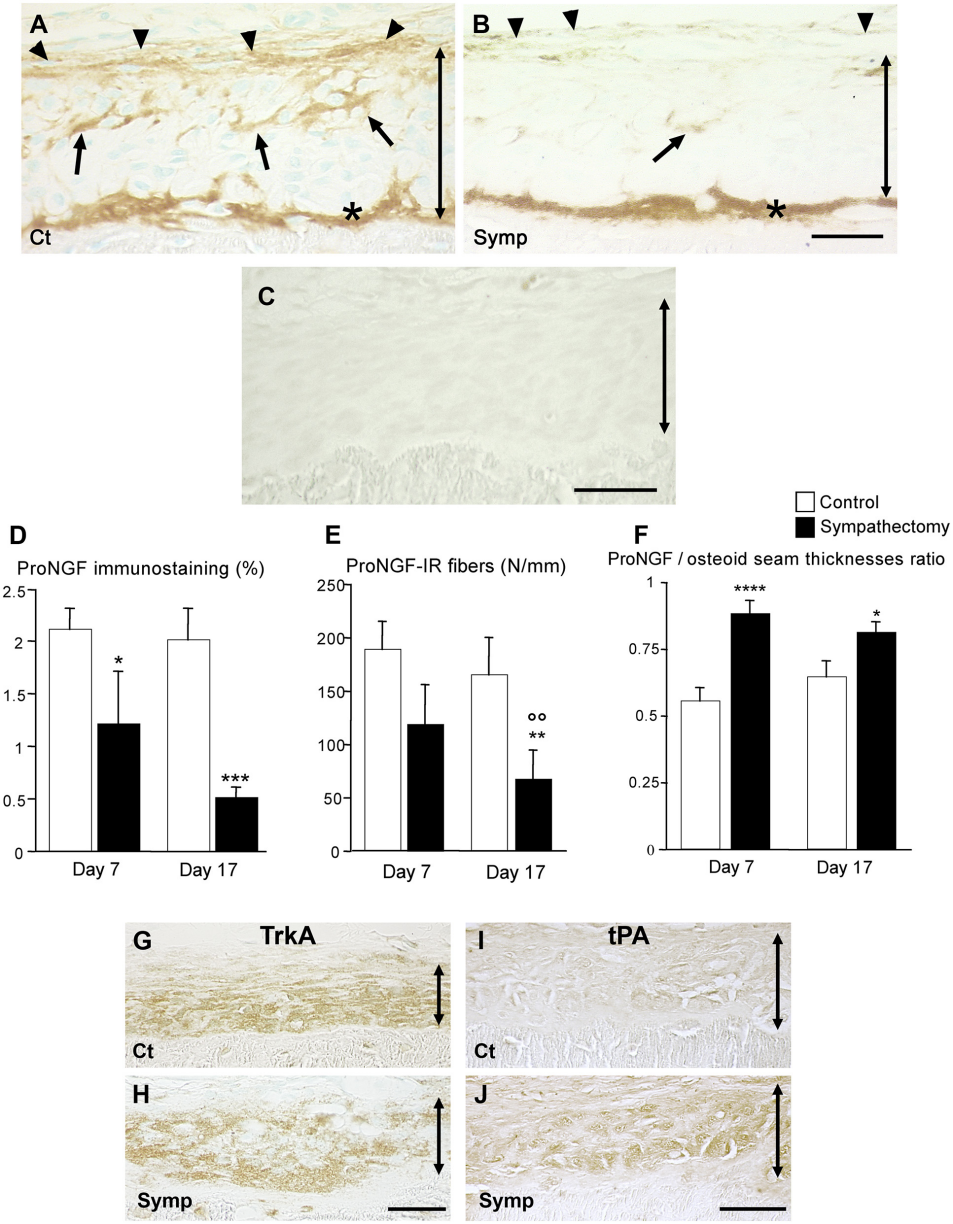
Effect of sympathectomy on proNGF, TrkA and tPA in the osteogenic compartment. **A**. 17-day control animal. proNGF is expressed extracellularly in the osteogenic compartment (arrows), within the osteoid seam (asterisk) and on the fibers at the periphery of the compartment (arrowheads). **B**. 17-day sympathectomy. The compartment is almost completely depleted in proNGF. Noticeably, the proNGF content of the osteoid seam was maintained in the sympathectomized animals (asterisk). Magnification bar (**A** and **B**): 50 μm. (**C**) Immunocontrol; no primary antibody used. Bar = 50 μm. Time-course changes in proNGF distribution in the osteogenic layer (**D**), in proNGF-positive fibers (**E**), and in proNGF/osteoid seam thicknesses ratio (**F**). * *P*<0.05, **P≤0.02, ***P<0.01, ****P<0.005 vs the corresponding controls. °° *P*<0.02 vs the 7-days sympathectomized animals. Values are mean ± SEM. Effects after 17 days of sympathectomy on TrkA (**G** and **H**) and tPA (**I** and **J**) immunostainings. Magnification bars: 50 μm. Ct: control animal; Symp: sympathectomized animal. The double arrows delineate the osteogenic compartment.

In the osteogenic compartment, only proNGF was expressed. The immunostaining was located in the intercellular spaces of the osteogenic layer and a portion of the osteoid seam was strongly immunostained (Fig 2A). Fibrous structures were immunostained at the boundary between the osteogenic and non-osteogenic compartments (Fig 2A), they correspond to a network of VIP-IR fibers [21,23]. At 7 days, sympathectomy already affected the osteogenic layer. On day 17, proNGF staining had almost completely vanished, except in the osteoid seam (Fig 2B). The morphometric analysis showed that sympathectomy decreased the percentage of proNGF immunostaining in the osteogenic layer on days 7 and 17 (-41%, p<0.05 and -65%, p<0.01 vs the corresponding controls, respectively. Fig 2D); however the two sympathectomy groups did not significantly differ. The number of immunoreactive fibers was decreased on day 17 (-60%, p = 0.02 vs the corresponding controls, Fig 2E); the two sympathectomy groups were significantly different (p<0.02). Sympathectomy reduced the thickness of the osteoid seam on day 17 (ct: 6.3 ± 0.3 μm, sympathectomy: 4.9 ± 0.3 μm, p<0.05), however the fraction of the osteoid seam positive for proNGF increased in sympathectomized animals at both time points (+ 60%, p<0.005 on day 7 and + 22%, p<0.05 on day 17, respectively, Fig 2F). TrkA was expressed in the osteogenic layer; its expression was not modified in sympathectomized animals (Fig 2G and 2H).

#### Tissue Plasminogen Activator expression

proNGF can be extracellularly cleaved in ßNGF by plasmin activated by tPA and selective metalloproteinases [25,30]. To assess whether proNGF may be processed into its mature form in the osteogenic layer, immunostaining for tPA was performed. tPA was not expressed in the control animals (Fig 2I); in contrast tPA was expressed in 17-days sympathectomized animals (Fig 2J).

#### Semaphorin3a and neuropilin-1 expressions

**Fig 3:**
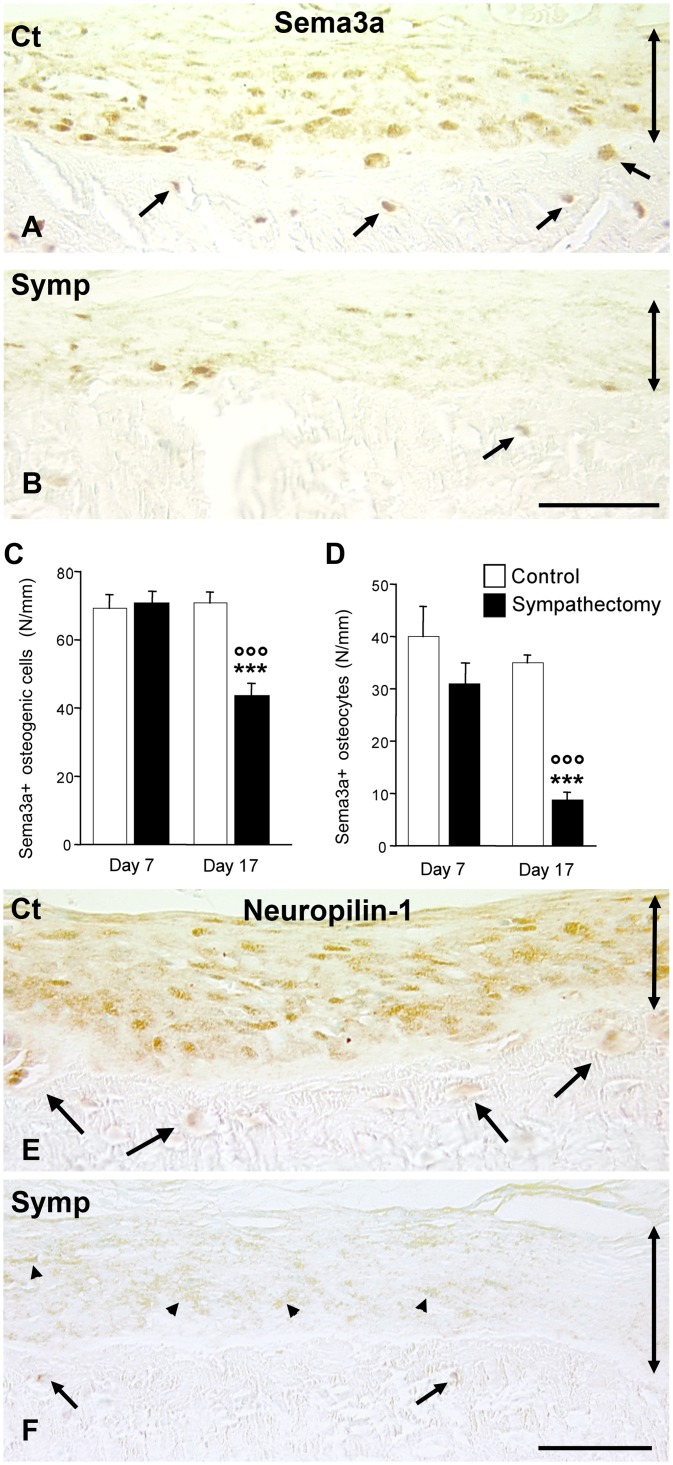
Semaphorin3a expression in the osteogenic compartment. **A**. 17-day control. Most cells were immunopositive in the osteogenic layer. Osteocytes subjacent to the cortical surface also expressed Sema3a (arrows). **B**. 17-day sympathectomy. Only a few scarce cells expressed the marker, most osteocytes were no longer immunopositive. Magnification bar (**A** and **B**): 50 μm. **C** and **D**: Changes with time in Sema3a-positive osteogenic cells (**C**) and osteocytes (**D**). *P<0.05, ***P<0.01 vs the corresponding controls. °°° *P*<0.002 versus the 7-day sympathectomy group. Immunostaining for neuropilin-1 in a control animal **(E)** and in a 17-days sympathectomized animal (**F**). Sympathectomy strongly reduced receptor expression. Arrowheads point out the osteogenic cells and arrows, the osteocytes. Magnification bar (**E** and **F**): 50 μm. Ct: control animal; Symp: sympathectomized animal. The double arrows delineate the osteogenic compartment.

Sema3a was expressed by the osteogenic cells and by the osteocytes close to the bone surface to a distance of about 60 μm (Fig 3A). The number of osteogenic sema3a^+^ cells decreased 17 days after sympathectomy (-39%, p<0.001 vs corresponding controls. Fig 3B and 3C). The drop in immunopositive osteocytes was strong (-76%; p<0.001 vs corresponding controls. Fig 3B and 3D). For the two cell types, the sympathectomy groups were significantly different (p<0.002). The distribution of NRP1 was similar to that of sema3a in the controls (Fig 3E). Its expression was reduced in 17-days sympathectomized animals and osteocytes did not express the marker anymore (Fig 3F).

Overall, these data indicate that the cholinergic nervous system controls proNGF storage in the extracellular matrix and sema3a expression by osteogenic cells and osteocytes. Sympathectomy depleted the osteogenic compartment of the two factors. tPA expression in sympathectomized animals was associated with proNGF depletion.

### Effects of sympathectomy in the non-osteogenic compartment

**Fig 4:**
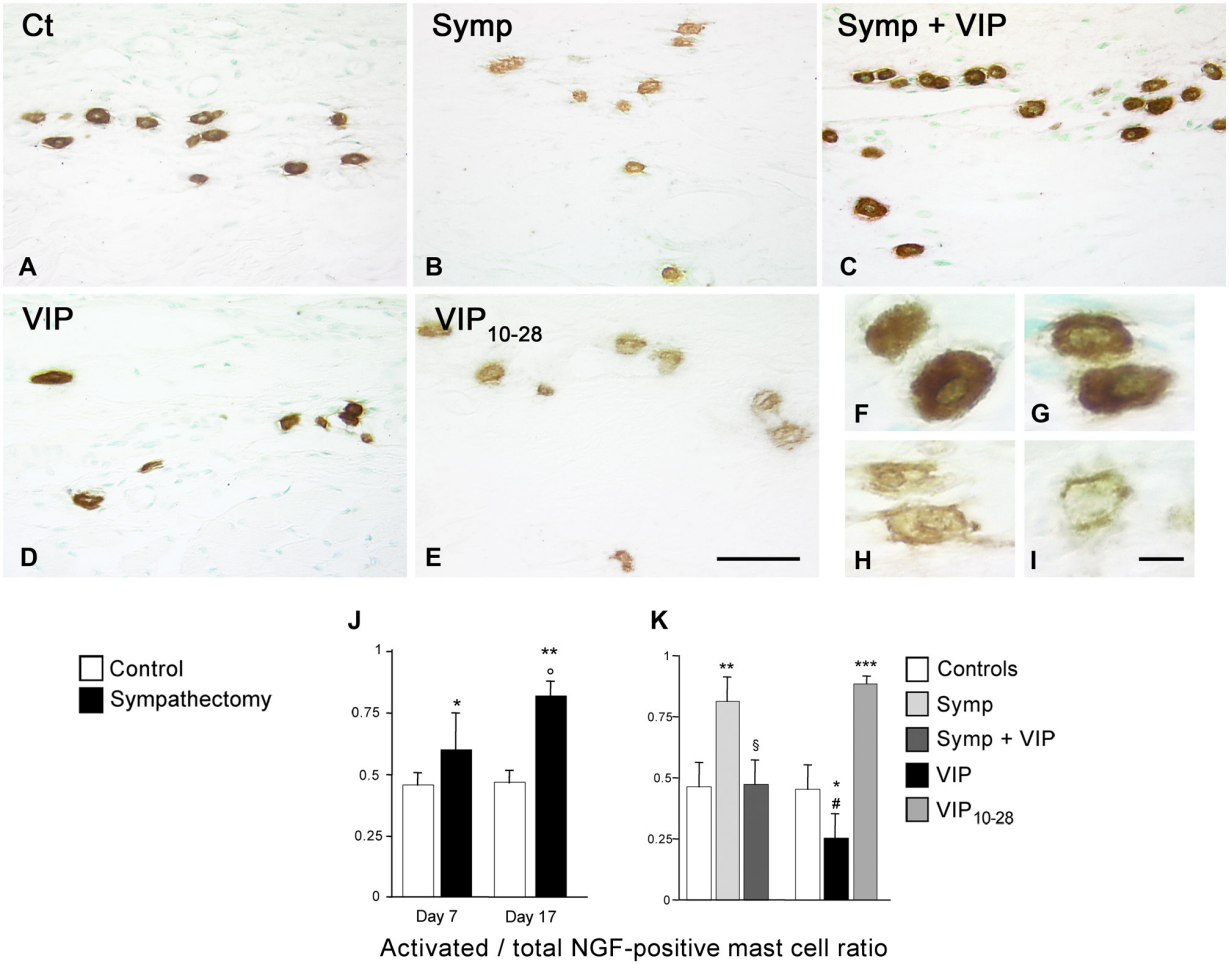
ßNGF expression. Only mast cells in the non-osteogenic compartment were immunostained. **A.** Control animal. **B.** 17-day sympathectomy. VIP treatment of sympathectomized (**C**) or intact (**D**) animals. VIP_10-28_ treatment (**E**). Sympathectomy caused mast cell activation and ßNGF release, as did VIP_10-28_ treatment. VIP treatments stabilized the cells. **F** to **I.** Different stages of mast cell activation and ßNGF depletion. When mast cells are activated, their granule content is released outside the cell, thus the immunostaining for the marker progressively decreases within the cell. Magnification bars, **A** to **E**: 50 μm; **F** to **I**: 10 μm. Variations in ßNGF-positive activated/total mast cells ratio, **J**: time-related changes and **K**: effect of the different treatments on the activated/total mast cells ratio. *P<0.05, ** *P*<0.01, ***P<0.005 vs the corresponding controls. ° *P*<0.05 vs the 7-day sympathectomy. § *P*<0.01 vs sympathectomy. # *P*<0.005 vs VIP_10-28_ group. Values are mean ± SEM.

ßNGF was expressed only in mast cells residing away from the bone surface close to the vessels irrigating the site; this population is involved in the control of osteoclast precursor entry in the periosteum [29,31]. The identification of the immunopositive cells as mast cells was confirmed by comparing adjacent sections stained with toluidine blue (pH 3.8). In the controls, mast cells were evenly and strongly immunostained (Fig 4A and 4F) whereas in sympathectomized animals (Fig 4B) most mast cells were partially empty, indicating cell activation (Fig 4G–4I). The total number of mast cells in the site did not vary. While the activated mast cells/total mast cells ratio remained constant in the control groups, it increased after sympathectomy (+76%, p<0.01 on day 17 vs their controls; Fig 4J).

Thus, the destruction of the sympathetic system induces mast cell activation and ßNGF release in the extracellular milieu, suggesting that factors synthesized by the sympathetic fibers stabilize mast cells.

### Effects of VIP and VIP receptor antagonist

#### Effect of VIP on sympathectomized animals

**Fig 5:**
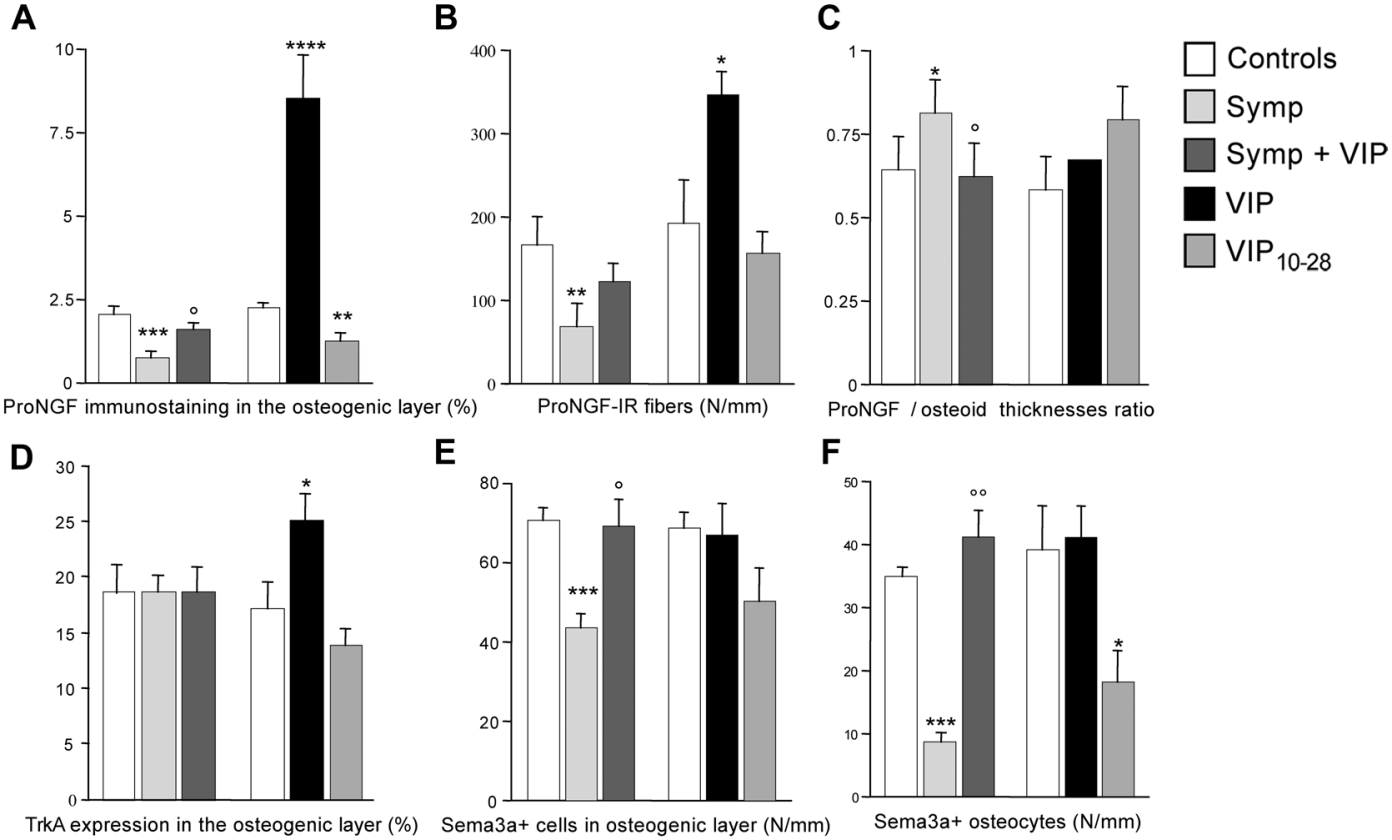
Effect of VIP treatments or VIP antagonism. Expression of proNGF in the osteogenic layer **(A)**, in peripheral fibers **(B)**, on proNGF/osteoid thicknesses ratio **(C)**. Expressions of TrkA (**D)** and of semaphorin 3a in the osteogenic cells (**E)** and the osteocytes **(F)**. *P<0.05, **P≤0.02, ***P <0.01, ****P<0.002 vs the corresponding controls. °*P*<0.01, °°*P*<0.001 vs sympathectomy. Values are mean ± SEM.

When sympathectomized animals were treated with VIP for 10 days, proNGF expression in the osteogenic layer increased to the level of the control animals (2.2x, p<0.05 vs sympathectomized animals. Fig 5A) while the fiber network immunostaining did not increase significantly (Fig 5B). The proNGF thickness/osteoid thickness ratio was restored (-23%, p<0.05 vs sympathectomized animals; Fig 5C). VIP treatment restored the number of osteogenic cells and osteocytes expressing sema3a (+60%, p<0.01 and +500%, p<0.001 vs sympathectomized group, respectively; Fig 5E and 5F). Similarly, NRP1 expression was recovered. Like sympathectomy, VIP treatment had no effect on TrkA expression (Fig 5D).

In the non-osteogenic compartment, the ßNGF^+^ activated/total mast cell ratio returned to control value (-42%, p<0.01 vs sympathectomized animals; Fig 4C and 4K).

Thus, VIP treatment of sympathectomized rats fully restored NGF and sema3a expressions despite the destruction of the cholinergic fibers, suggesting a role for VIP in the behavior of periosteum cell populations.

#### Effects of VIP and VIP_10-28_ in intact animals

Treating intact rats with VIP increased proNGF expression, the immunopositive fiber network (4x, p<0.002 vs controls and 1.8x p<0.05 vs controls, respectively; Fig 5A and 5B) and TrkA expression (+46%, p<0.05 vs controls, Fig 5D) in the osteogenic compartment. No tPA expression was observed (not shown). VIP treatment had no additive effect on sema3a (Fig 5E and 5F) and NRP1 expressions.

VIP_10-28_ treatment decreased the expression of proNGF (-45%; p<0.02 vs controls; Fig 5A), but did not modify TrkA expression. Like in sympathectomized animals, tPA was expressed (not shown). VIP_10-28_ treatment decreased the number of immunopositive osteocytes (-54%, p<0.05 vs controls; Fig 5F). NRP1 expression was decreased as well.

The osteoid parameters were not significantly affected by the two treatments (Fig 5C).

In the non-osteogenic compartment, VIP treatment decreased the ßNGF^+^ activated/total mast cell ratio (-44%; p<0.05 vs the controls) while VIP_10-28_ treatment doubled it (p<0.005 vs the controls; Fig 4D, 4E and 4K).

### Changes in CGRP expression

**Fig 6:**
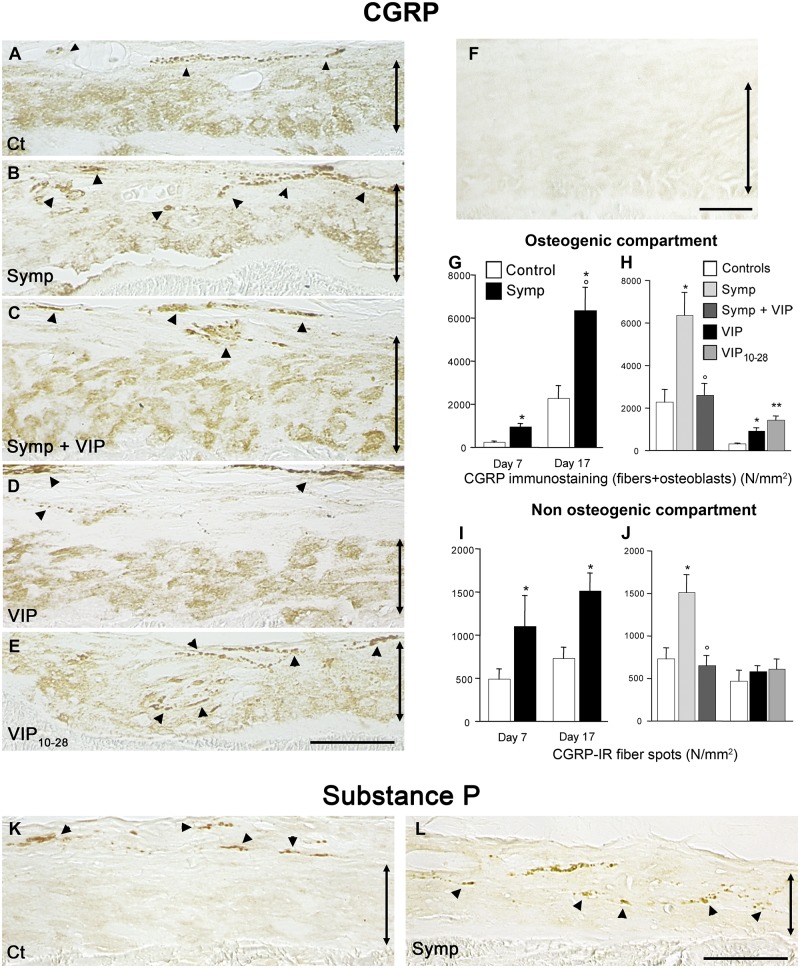
Variations in CGRP-immunostaining and expression. Immunoreactive sensory fibers and osteogenic cells expressed CGRP. **A**. 17-day control animal (Ct). CGRP fibers (arrowheads) were located at the boundary between the two compartments. **B**. Day-17 sympathectomized animal (Symp). **C**. VIP-treated sympathectomized animal (Symp+VIP). **D**. VIP-treated intact animal (VIP). **E**. VIP_10-28_-treated animal (VIP_10-28_). Same magnification from **A** to **E,** bar: 50 μm. **(F)** Immunocontrol; no primary antibody used. Bar = 50 μm. In the osteogenic compartment, CGRP expression increased as soon as day 7 in the sympathectomy group, and continued so at day 17 **(G)**. VIP treatment of sympathectomized animals restored the expression profile of controls. Treating intact animals with VIP_10-28_ or VIP increased CGRP expression **(H**). In the non-osteogenic compartment, sympathectomy increased sensory fibers **(I**). Effects of treatments on the sympathectomized and intact animals on CGRP-IR expression **(J)**. *P<0.05, **P<0.01 vs the corresponding controls. °*P*<0.05 vs the sympathectomized animals. # *P*<0.05 vs the VIP-treated cultures. Values are mean ± SEM. Immunostaining for substance P, another marker of sensory fibers. **K.** Control animal. **L.** Sympathectomized animal. Same magnification in **K** and **L,** bar: 50 μm. The double-headed arrows delineate the osteogenic compartment.

In the control group, CGRP-IR fibers appeared as isolated or aligned dense dots, they were located in the non-osteogenic compartment, some of them present at the boundary of the osteogenic compartment (Fig 6A). The osteogenic cells were also immunopositive for CGRP. It must be pointed out that we could not morphometrically discriminate the two sources of CGRP so that the quantitative data included both fiber and osteoblast CGRP expressions.

Sympathectomy resulted in the penetration of CGRP-IR fibers in the osteogenic compartment (Fig 6B) but in a weaker immunostaining of the osteogenic cells. Quantitatively, the immunostaining increased from day 7 to day 17 in the sympathectomized animals (7x; p<0.01 vs 7-day sympathectomized animals. Fig 6G). Treating sympathectomized animals with VIP reversed CGRP-IR fiber growth; as in the controls, fibers were no longer present in the osteogenic compartment while the osteogenic cells re-expressed the marker (Fig 6C). The quantitative data showed a global reduction in CGRP expression (-60%, p<0.05 vs sympathectomized animals. Fig 6H). We confirmed sensory fiber sprouting in sympathectomized animals by performing immunostaining for substance P (Fig 6K and 6L).

VIP treatment of intact animals increased CGRP expression (3.5x, p<0.05 vs controls, Fig 6H). Noticeably, the positive fibers remained peripheral while the osteogenic cells strongly expressed the marker (Fig 6D). VIP_10-28_ treatment increased CGRP expression (5.6x, p<0.01 vs controls. Fig 6H). This increase was due to the sprouting of immunopositive fibers within the osteogenic compartment, CGRP expression in the osteogenic cells was weak (Fig 6E).

In the non-osteogenic compartment of the sympathectomized animals CGRP-IR fibers increased (p<0.05 vs corresponding controls at the two time points; Fig 6I). VIP treatment of sympathectomized animals normalized the number of CGRP-IR fibers (Fig 6J). In intact animals VIP and VIP_10-28_ treatments did not change the numbers of CGRP-IR fibers (Fig 6J).

## Discussion

In this study the communications between the osteogenic cells and the nervous fibers innervating the mandible periosteum were evaluated by investigating the expression by the osteogenic lineage of NGF, a nerve attractant molecule, and sema3a, a repulsive molecule, which are involved both in the maintenance of the sympathetic and sensory fiber networks and in local bone homeostasis [19,32]. In this periosteum, sema3a may physiologically prevent VIP-IR fiber penetration within the osteogenic compartment, an exclusion process reported in the central nervous system [33]. Moreover, since sensory CGRP-IR fibers are positioned only outside the compartment, sema3a may also hinder their penetration. The loss of VIP-expressing fibers after sympathectomy disorganized this molecular network as proNGF and sema3a expressions were strongly reduced, and induced the sprouting of sensory fibers in the osteogenic compartment. The implication of the neuropeptide VIP in periosteum equilibrium may be supported by the effects of VIP treatment of sympathectomized animals that fully restored periosteum homeostasis, and of VIP_10-28_ treatment of intact animals that mimicked most consequences of sympathectomy.

### NGF in the periosteum

NGF, a trophic factor for nerve fibers, is also involved in the differentiation and survival of the osteogenic cells [10,12,34]. In this study we differentiated the two different forms of NGF, namely the proform, proNGF, and the mature form, ßNGF. Their expressions were well segregated: proNGF was released and stored in the extracellular matrix of the osteogenic compartment while ßNGF was only expressed in the non-osteogenic compartment.

Outside the nervous system, many cell types release proNGF that has paracrine and autocrine functions [35]. As NGF is involved in osteoblastic lineage survival, one can expect that proNGF depletion resulted in increased cell death in the osteogenic compartment. Instead, the rate of apoptosis remains stable in the periosteum after sympathectomy as did the thickness of the ALP layer [21]. As proNGF can be extracellularly cleaved in ßNGF by plasmin and metalloproteinases [25,30], we looked for tPA expression. We found that tPA was expressed in the osteogenic layer of sympathectomized and VIP_10-28_-treated animals only. Noticeably, treating sympathectomized animals with VIP suppressed tPA expression and restored the proNGF pool in the extracellular matrix. This suggests a processing of proNGF in ßNGF by the osteogenic cells in case of VIP inactivation. We hypothesize that the osteogenic cells metabolized the proNGF stored in the extracellular matrix for their survival and that they continued to synthesize the factor, probably at a lower rate since NGF is secreted in concentration proportional to nerve fiber density [9]. The stability of TrkA expression in sympathectomized animals supports this hypothesis. However, no ßNGF immunostaining was observed in this compartment; in fact ßNGF is rapidly degraded in the extracellular environment by plasmin and MMP-9 [30]. Interestingly, mouse osteoblasts expressed MMP-9 mRNA when treated with VIP_10-28_ (S1 File and Figure F in S1 File). The osteoid seam contained a band of proNGF that was not affected by sympathectomy or VIP_10-28_ treatment, suggesting that once embedded in the osteoid tissue, it is no longer accessible to environmental degradation.

ßNGF was only expressed by the mast cells residing in the non-osteogenic compartment. Connective tissue mast cells synthesize, store in secretion vesicles and constitutively secrete ßNGF [36]. As these mast cells reside close to the sensory fibers, ßNGF secretion may play a role in the maintenance of the sensory network. Since sympathectomy activated mast cell degranulation, and thus enhanced ßNGF release, this disequilibrium may in turn cause the increase in sensory fibers occurring after sympathectomy [21,37]. Although VIP_10-28_ treatment increased mast cell activation, CGRP-IR fibers did not increase in the non-osteogenic compartment. The intact sympathetic network of these animals likely prevented sensory fiber growth.

In summary, our data support the hypothesis that NGF has various roles in periosteum metabolism. Moreover a strict compartmentalization of the NGF forms appears to regulate periosteum sensory and sympathetic nervous system homeostasis, with proNGF being associated with the sympathetic system and ßNGF controlling the sensory system. In addition different cell types are responsible for this dichotomy.

### Semaphorin 3a and periosteum metabolism

Sympathectomy and VIP_10-28_ treatment reduced the expressions of sema3a and of its receptor NRP1 in the osteogenic compartment. Their recovery in the sympathectomized animals treated with VIP highlighted the role of VIP on sema3a metabolism. Beside its repulsive role on sympathetic and sensory fiber networks, sema3a promotes osteoblast differentiation [19]. However, Fukuda et al. showed that sensory fiber-derived, and not osteogenic cell-derived sema3a, influenced osteoblast metabolism [38]. The sprouting of sensory fibers in the sympathectomized and VIP_10-28_-treated animals may contribute to osteogenic cell survival by providing nerve-derived sema3a. In addition, sensory fibers also release CGRP that is trophic for osteoblasts [39,40].

Sema3a has a bifunctional effect on bone metabolism: besides its action on osteogenic cells, it inhibits osteoclast differentiation upstream RANKL and is repellent for osteoclast precursors [19]. In this view, the osteocytes close to the bone surface expressing sema3a may prevent access to the bone surface and protect from unnecessary resorption by keeping osteoclast precursors at distance and hindering osteoclast formation. One could therefore expect an increase in periosteum preosteoclasts and a stimulation of resorption in the sympathectomized animals; instead, sympathectomy results in depletion of TRAP^+^ preosteoclasts and resorption inhibition [21,23]. In fact, we previously found that sympathectomy blocked the traffic to the bone surface of ED1^+^ cells that are osteoclast precursors [28], at the boundary of the osteogenic compartment where they accumulated [21]. We also found that OPG mRNA expression increased in the whole mandible periosteum of sympathectomized animals without change in RANKL mRNA [21]. Accordingly, OPG mRNA expression was increased by mouse osteoblasts treated with VIP_10-28_ (S1 File and Figure I in S1 File). Thus the loss of sema3a repulsive effect was likely balanced by a RANKL/OPG ratio unfavorable to resorption. This anti-resorption effect was further enhanced by the release of CGRP, an inhibitor of osteoclast differentiation and bone resorption [40], by the osteogenic cells and the sensory fibers sprouting in the osteogenic compartment as a consequence of sema3a depletion. Thus while VIP regulates sema3a expression by osteogenic cells, sema3a may restrain and counterbalance the pro-resorption action of VIP [41], in synergy with CGRP whose expression by osteogenic cells vary with VIP release or inactivation.

**Fig 7:**
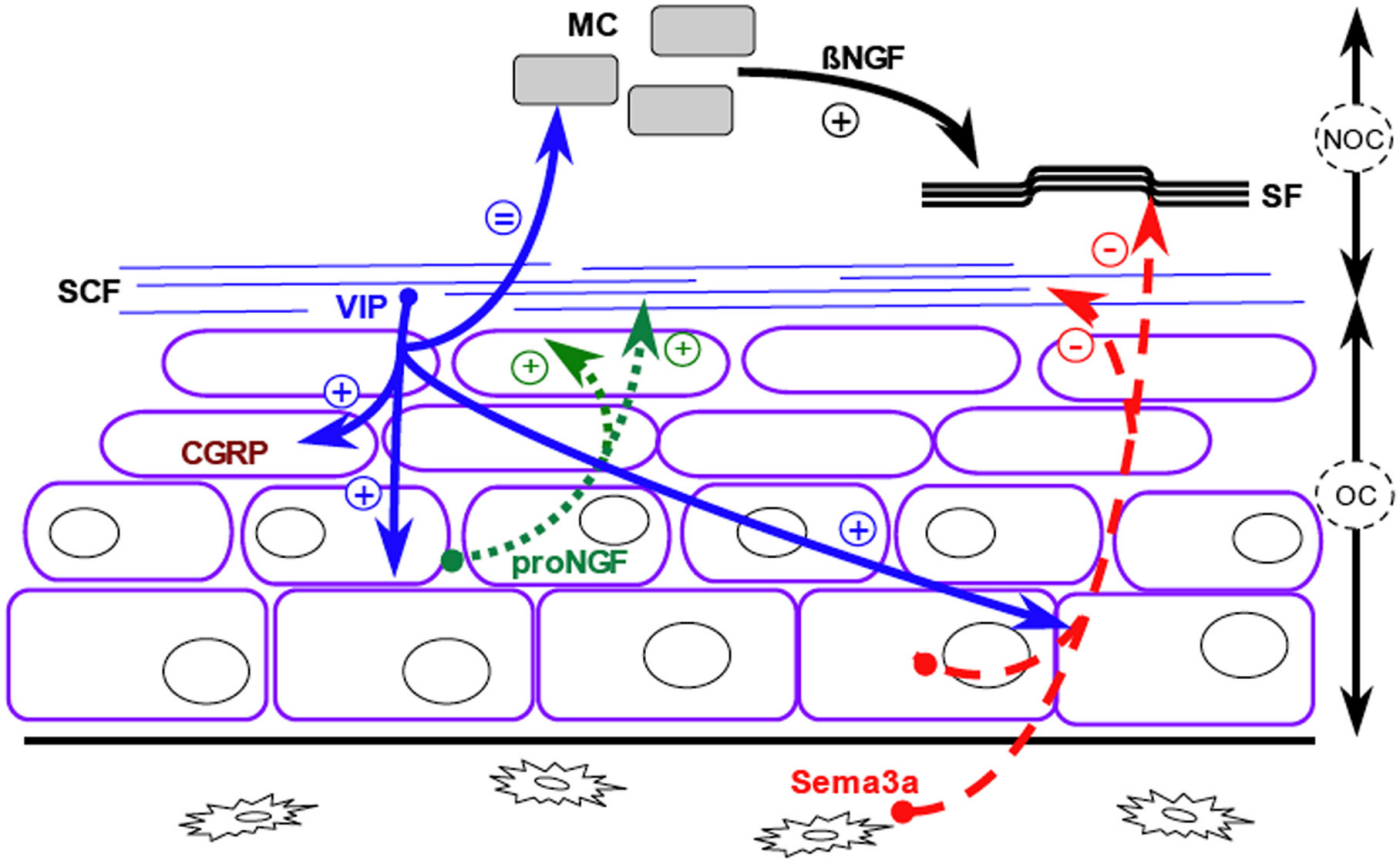
Schematic representation of the crosstalk between nerve fibers and osteogenic cells in the periosteum. VIP (blue solid arrows) synthesized by the sympathetic cholinergic fibers (SCF) induces the release by the osteogenic cells (purple outline) of proNGF (green dotted arrows), which is stored in the extracellular matrix. proNGF has a trophic (+) effect on sympathetic fibers and osteogenic cells. VIP also induces the release by osteogenic cells and osteocytes of semaphorin 3a (red broken arrows) that repulses (-) sympathetic and sensory fibers (wavy lines, SF) out the osteogenic compartment. VIP stabilizes (=) mast cells (MC, grey filling) to prevent excess release of ßNGF, which is trophic for sensory fibers. VIP induces CGRP (brown) expression by osteogenic cells. VIP unavailability after sympathectomy or VIP_10-28_ treatment disrupted this metabolic network, favored sensory fiber penetration in the osteogenic compartment, decreased proNGF and semaphorin 3a synthesis, enhanced ßNGF release by mast cells and sprouting of sensory fibers in the non-osteogenic compartment. The double arrows delineate the osteogenic compartment (OC) and the non-osteogenic compartment (NOC).

In conclusion, interactions between sympathetic fibers and osteogenic cells in the mandible periosteum, besides their trophic effect on nervous fibers, locate the VIP-IR fibers at the periphery of the osteogenic layer and prevent its penetration by sensory fibers (Fig 7). In this site, VIP-elicited expressions of NGF and sema3a participate to the trophic maintenance of the osteogenic cells and to the prevention of hazardous resorption by possibly regulating the number of preosteoclasts and by protecting the bone surface by repelling them. Nevertheless the membranous and neurectoderm origin of the mandible prevents a direct transposition to the behavior of the periosteum of the appendicular and axial skeletons that are of endochondral and mesoderm origin.

## Supporting Information

S1 File Effects of VIP and VIP_10-28_ on mRNA expression of selected markers. Primary osteoblasts were isolated from calvariae of 2- to 3- day-old mixed C57BL/6 mice (Charles River, L’Arbresle, France), expanded for 5 days and re-plated in differentiation medium [growth medium supplemented with 50 μM ascorbic acid, 10 nM dexamethasone and 5 mM beta-glycerophosphate (Sigma St Louis, MO, USA)]. Cells were seeded at 1x10^5^ cells/well in 6-well-plates. At day 7 (early treatment) and 18 (late treatment), cells were treated with VIP (1 μM, NeoMPS, Strasbourg, France) or VIP_10-28_ (1 μM, American Peptide Company, Sunnydale, CA, USA) for 72 hours. Untreated cells were used as controls. Treatment protocols (concentrations, frequency and duration) were determined after Persson and Lerner (2005). Total RNA from osteoblasts stopped at days 10 and 21 was isolated. The primers used were: *Alp*: F5’-3’ GATATCGACGTGATCATGGG, R5’-3’ CATCCAGTTCGTATTCCACA; *Collagen 1A1*: F5’-3’ TGACTGGAAGAGTGGAGAGTA, R5’-3’ TCTTGCTGATGTACCAGTTCT; *Ngf*: F5’-3’ AAGGGCAAGGAGGTGACAGT, R5’-3’ GCTCGGCACTTGGTCTCAAA; *Semaphorin 3a*: F5’-3’ AAAACGGTCGTGGGAAGAGC, R5’-3’ TCCGCAGCAGTTCCAGAGTA; *tPa*: F5’-3’ CTCCTGGAGAGAGATTCCTT, R5’-3’ CTGTATGTTCTGCCCAAGAC; *Mmp9*: F5’-3’ GGTGATCTCTTCTAGAGACTGG, R5’-3’ CTAAAGTAGCTGGAAAAGGTT; *Cgrp*: F5’-3’ ACTGGTGAGGACTATATGC, R5’-3’ GTTGCAGGATCTCTTCTGAG; *Opg*: F5’-3’ GCTTATCAGAGCCTCATCAC, R5’-3’ GGTCCAACTACAGAGGAACA; *Rankl*: F5’-3’ GACTCATTTCGTGGAACATT, R5’-3’ AAAACCGTTGTGTAATCACC; *Gapdh*: F5’-3’ TGTGTCCGTCGTGGATCTGA, R5’-3’ TTGCTGTTGAAGTCGCAGGAG; *Actin*: F5’-3’ GTGGCATCCATGAAACTACAT, R5’-3’ GGCATAGAGGTCTTTACGG. Quantitative real-time PCR analysis was carried out. Relative gene expression levels were estimated using the deltaCP method (Pfaffl, 2001). *Gapdh* and *Actin* were used as housekeeping genes for normalization (Roche et al., 1992). For all assays, at least 3 wells were used. Experiments were independently reproduced three times. Expression of the osteoblastic differentiation markers collagen 1a1 (Figure A in S1 File) and ALP (Figure B in S1 File) was first assessed to confirm the phenotype. VIP increased the expressions of ALP and collagen 1a1 mRNAs (both p<0.05) while VIP_10-28_ had no effect (Figures C and D in S1 File). The treatments had no effect on NGF mRNA (early or late treatments) (Figure E in S1 File). tPA mRNA expression was not modified by VIP but was augmented by VIP_10-28_ (3.6x, p<0.005 vs the control cultures, early treatment) (Figure F in S1 File). VIP decreased the expression of MMP-9 mRNA that controls NGF degradation in the extracellular environment (-33%, p<0.05, late treatment) while VIP_10-28_ strongly increased it (5.3x, p<0.05) (Figure G in S1 File). VIP had no effect on sema3a expression, while VIP_10-28_ decreased it (-70%, p<0.05, late treatment) (Figure H in S1 File). As sema3a behaves as a repellent factor on osteoclast precursors (Hayashi et al., 2012), we verified whether VIP and VIP_10-28_ affected the expression of OPG and RANKL that are pivotal for osteoclast differentiation and activity. VIP reduced OPG expression (-29%, p<0.05, early treatment) while VIP_10-28_ increased it (p<0.05, early and late treatments) (Figure I in S1 File). VIP strongly increased RANKL expression (9x, p<0.05, early and late treatments) whereas VIP_10-28_ had no effect (Figure J in S1 File). VIP increased (+50%, p<0.05 vs the controls) and VIP_10-28_ decreased CGRP expression (-40%, p<0.05 vs the controls), both during the late treatment (Figure K in S1 File).
(DOC)

S1 Fig Effects of VIP and VIP_10-28_ on mRNA expression of selected markers by mouse calvaria primary osteoblasts. Results are mean ± SEM of 3 different experiments. *P<0.05, **P<0.005 vs the untreated control cultures; °*P*<0.05 vs the VIP-treated cultures. Persson E, Lerner UH. The neuropeptide VIP potentiates IL-6 production induced by proinflammatory osteotropic cytokines in calvarial osteoblasts and the osteoblastic cell line MC3T3-El. *Biochem Biophys Res Commun*. 2005;335: 705–711. Pfaffl MW. A new mathematical model for relative quantification in real-time RT-PCR. *Nucleic Acids Res*. 2001;29: e45. Roche PC, Ryan RJ, McCormick DJ. Identification of hormone-binding regions of the luteinizing hormone/human chorionic gonadotropin receptor using synthetic peptides. *Endocrinology* 1992;131: 268–274.
(TIF)

## Abbreviations

AdB2R: beta-2 adrenergic receptors
ALP: alkaline phosphatase
CGRP: Calcitonin Gene-Related Peptide
IR: immunoreactive
NGF: Nerve Growth Factor
NRP1: Neuropilin-1
OPG: Osteoprotegerin
PBS: phosphate buffered saline
RANK: Receptor Activator of NF KappaB
RANKL: Receptor Activator of NF KappaB Ligand
Sema3a: Semaphorin 3A
tPA: tissue Plasminogen Activator
TrkA: Tyrosine kinase receptor A
VIP: Vasoactive Intestinal Peptide
