# Supplementary material for: Periosteum Metabolism and Nerve Fiber Positioning Depend on Interactions between Osteoblasts and Peripheral Innervation in Rat Mandible
Source: PLoS One. 2015 Oct 28;10(10):e0140848. doi: 10.1371/journal.pone.0140848 (PMC4624798; doi:10.1371/journal.pone.0140848)
Supplement: S1 File — Primary osteoblasts were isolated from calvariae of 2- to 3- day-old mixed C57BL/6 mice (Charles River, L’Arbresle, France), expanded for 5 days and re-plated in differentiation medium [growth medium supplemented with 50 μM ascorbic acid, 10 nM dexamethasone and 5 mM beta-glycerophosphate (Sigma St Louis, MO, USA)]. Cells were seeded at 1x10^5^ cells/well in 6-well-plates. At day 7 (early treatment) and 18 (late treatment), cells were treated with VIP (1 μM, NeoMPS, Strasbourg, France) or VIP10-28 (1 μM, American Peptide Company, Sunnydale, CA, USA) for 72 hours. Untreated cells were used as controls. Treatment protocols (concentrations, frequency and duration) were determined after Persson and Lerner (2005). Total RNA from osteoblasts stopped at days 10 and 21 was isolated. The primers used were: Alp: F5’-3’ GATATCGACGTGATCATGGG, R5’-3’ CATCCAGTTCGTATTCCACA; Collagen 1A1: F5’-3’ TGACTGGAAGAGTGGAGAGTA, R5’-3’ TCTTGCTGATGTACCAGTTCT; Ngf: F5’-3’ AAGGGCAAGGAGGTGACAGT, R5’-3’ GCTCGGCACTTGGTCTCAAA; Semaphorin 3a: F5’-3’ AAAACGGTCGTGGGAAGAGC, R5’-3’ TCCGCAGCAGTTCCAGAGTA; tPa: F5’-3’ CTCCTGGAGAGAGATTCCTT, R5’-3’ CTGTATGTTCTGCCCAAGAC; Mmp9: F5’-3’ GGTGATCTCTTCTAGAGACTGG, R5’-3’ CTAAAGTAGCTGGAAAAGGTT; Cgrp: F5’-3’ ACTGGTGAGGACTATATGC, R5’-3’ GTTGCAGGATCTCTTCTGAG; Opg: F5’-3’ GCTTATCAGAGCCTCATCAC, R5’-3’ GGTCCAACTACAGAGGAACA; Rankl: F5’-3’ GACTCATTTCGTGGAACATT, R5’-3’ AAAACCGTTGTGTAATCACC; Gapdh: F5’-3’ TGTGTCCGTCGTGGATCTGA, R5’-3’ TTGCTGTTGAAGTCGCAGGAG; Actin: F5’-3’ GTGGCATCCATGAAACTACAT, R5’-3’ GGCATAGAGGTCTTTACGG. Quantitative real-time PCR analysis was carried out. Relative gene expression levels were estimated using the deltaCP method (Pfaffl, 2001). Gapdh and Actin were used as housekeeping genes for normalization (Roche et al., 1992). For all assays, at least 3 wells were used. Experiments were independently reproduced three times. Expression of the osteoblastic differentiation markers collagen 1a1 (Figure A in S1 File) and ALP (Figure B in S1 File) was first assessed t [file pone.0140848.s001.doc]

**Supporting information**

**S1 File**

Primary osteoblasts were isolated from calvariae of 2- to 3- day-old mixed C57BL/6 mice (Charles River, L’Arbresle, France), expanded for 5 days and re-plated in differentiation medium [growth medium supplemented with 50 μM ascorbic acid, 10 nM dexamethasone and 5 mM beta-glycerophosphate (Sigma St Louis, MO, USA)]. Cells were seeded at 1x10^5^ cells/well in 6-well-plates. At day 7 (early treatment) and 18 (late treatment), cells were treated with VIP (1 μM, NeoMPS, Strasbourg, France) or VIP_10-28_ (1 μM, American Peptide Company, Sunnydale, CA, USA) for 72 hours. Untreated cells were used as controls. Treatment protocols (concentrations, frequency and duration) were determined after Persson and Lerner (2005).

Total RNA from osteoblasts stopped at days 10 and 21 was isolated. The primers used were: *Alp*: F5’-3’ GATATCGACGTGATCATGGG, R5’-3’ CATCCAGTTCGTATTCCACA; *Collagen 1A1*: F5’-3’ TGACTGGAAGAGTGGAGAGTA, R5’-3’ TCTTGCTGATGTACCAGTTCT; *Ngf*: F5’-3’ AAGGGCAAGGAGGTGACAGT, R5’-3’ GCTCGGCACTTGGTCTCAAA; *Semaphorin 3a*: F5’-3’ AAAACGGTCGTGGGAAGAGC, R5’-3’ TCCGCAGCAGTTCCAGAGTA; *tPa*: F5’-3’ CTCCTGGAGAGAGATTCCTT, R5’-3’ CTGTATGTTCTGCCCAAGAC; *Mmp9*: F5’-3’ GGTGATCTCTTCTAGAGACTGG, R5’-3’ CTAAAGTAGCTGGAAAAGGTT; *Cgrp*: F5’-3’ ACTGGTGAGGACTATATGC, R5’-3’ GTTGCAGGATCTCTTCTGAG; *Opg*: F5’-3’ GCTTATCAGAGCCTCATCAC, R5’-3’ GGTCCAACTACAGAGGAACA; *Rankl*: F5’-3’ GACTCATTTCGTGGAACATT, R5’-3’ AAAACCGTTGTGTAATCACC; *Gapdh*: F5’-3’ TGTGTCCGTCGTGGATCTGA, R5’-3’ TTGCTGTTGAAGTCGCAGGAG; *Actin*: F5’-3’ GTGGCATCCATGAAACTACAT, R5’-3’ GGCATAGAGGTCTTTACGG. Quantitative real-time PCR analysis was carried out. Relative gene expression levels were estimated using the deltaCP method (Pfaffl, 2001). *Gapdh* and *Actin* were used as housekeeping genes for normalization (Roche et al., 1992). For all assays, at least 3 wells were used. Experiments were independently reproduced three times.

Expression of the osteoblastic differentiation markers collagen 1a1 (A) and ALP (B) was first assessed to confirm the phenotype. VIP increased the expressions of ALP and collagen 1a1 mRNAs (both p<0.05) while VIP_10-28_ had no effect **(**C and D**)**. The treatments had no effect on NGF mRNA (early or late treatments) (E). tPA mRNA expression was not modified by VIP but was augmented by VIP_10-28_ (3.6x, p<0.005 vs the control cultures, early treatment) (F**).** VIP decreased the expression of MMP-9 mRNA that controls NGF degradation in the extracellular environment (-33%, p<0.05, late treatment) while VIP_10-28_ strongly increased it (5.3x, p<0.05) (G**).** VIP had no effect on sema3a expression, while VIP_10-28_ decreased it (-70%, p<0.05, late treatment) (H**).** As sema3a behaves as a repellent factor on osteoclast precursors (Hayashi et al., 2012), we verified whether VIP and VIP_10-28_ affected the expression of OPG and RANKL that are pivotal for osteoclast differentiation and activity. VIP reduced OPG expression (-29%, p<0.05, early treatment) while VIP_10-28_ increased it (p<0.05, early and late treatments) (I). VIP strongly increased RANKL expression (9x, p<0.05, early and late treatments) whereas VIP_10-28_ had no effect **(**J**)**. VIP increased (+50%, p<0.05 vs the controls) and VIP_10-28_ decreased CGRP expression (-40%, p<0.05 vs the controls), both during the late treatment **(**K**)**.

**Figure 1S.** **Effects of VIP and VIP_10-28_ on mRNA expression of selected markers by mouse calvaria primary osteoblasts.** Results are mean ± SEM of 3 different experiments. **P*<0.05, ***P*<0.005 vs the untreated control cultures; °*P*<0.05 vs the VIP-treated cultures.

Persson E, Lerner UH. The neuropeptide VIP potentiates IL-6 production induced by proinflammatory osteotropic cytokines in calvarial osteoblasts and the osteoblastic cell line MC3T3-El. *Biochem Biophys Res Commun.* 2005;335: 705-711.

Pfaffl MW. A new mathematical model for relative quantification in real-time RT-PCR. *Nucleic Acids Res.* 2001;29: e45.

Roche PC, Ryan RJ, McCormick DJ. Identification of hormone-binding regions of the luteinizing hormone/human chorionic gonadotropin receptor using synthetic peptides. *Endocrinology* 1992;131: 268-274.
