# Supplementary figures and images for: Periosteum Metabolism and Nerve Fiber Positioning Depend on Interactions between Osteoblasts and Peripheral Innervation in Rat Mandible
Source: PLoS One. 2015 Oct 28;10(10):e0140848. doi: 10.1371/journal.pone.0140848 (PMC4624798; doi:10.1371/journal.pone.0140848)

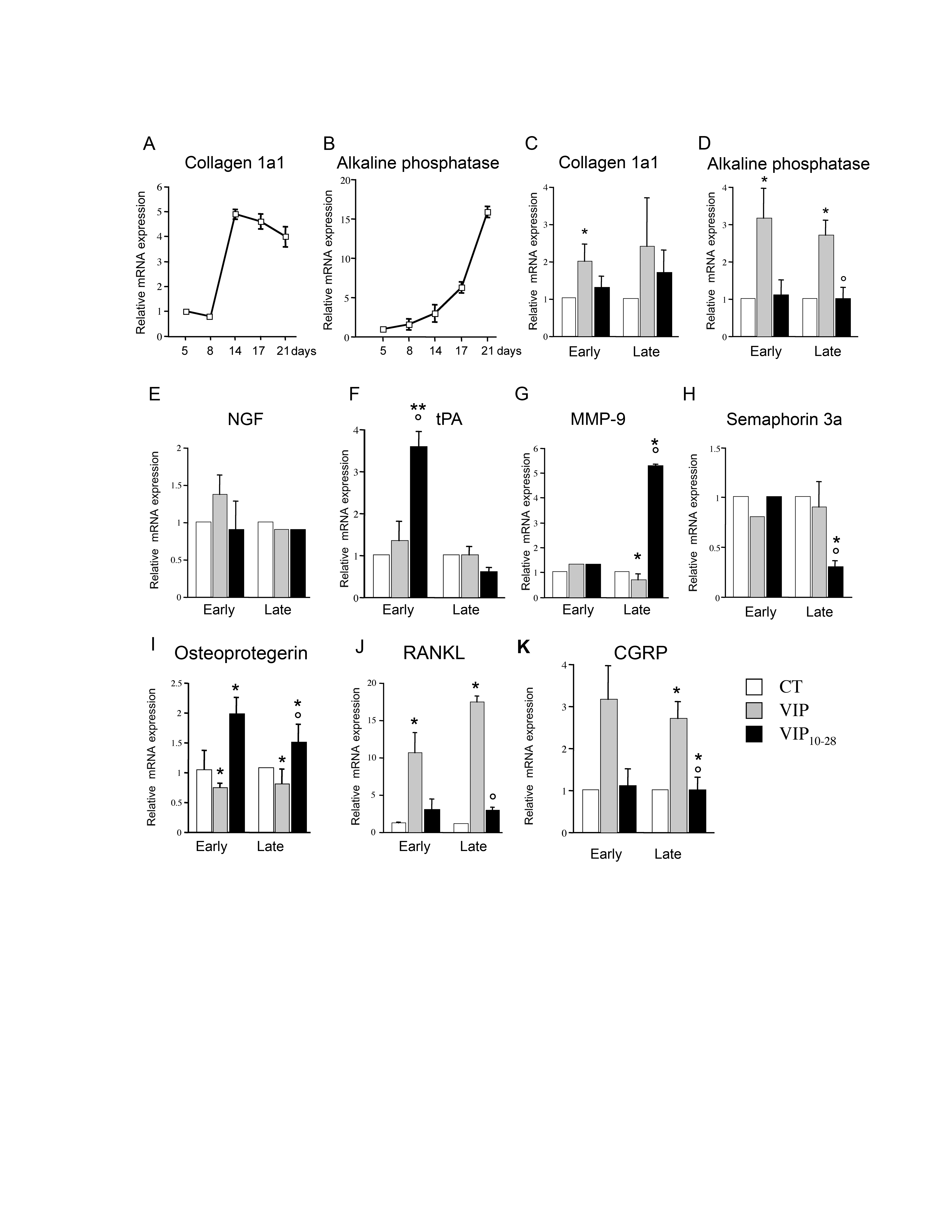

Supplement: S1 Fig — Results are mean ± SEM of 3 different experiments. *P<0.05, **P<0.005 vs the untreated control cultures; °P<0.05 vs the VIP-treated cultures. Persson E, Lerner UH. The neuropeptide VIP potentiates IL-6 production induced by proinflammatory osteotropic cytokines in calvarial osteoblasts and the osteoblastic cell line MC3T3-El. Biochem Biophys Res Commun. 2005;335: 705–711. Pfaffl MW. A new mathematical model for relative quantification in real-time RT-PCR. Nucleic Acids Res. 2001;29: e45. Roche PC, Ryan RJ, McCormick DJ. Identification of hormone-binding regions of the luteinizing hormone/human chorionic gonadotropin receptor using synthetic peptides. Endocrinology 1992;131: 268–274. (TIF) [file pone.0140848.s002.tif]
